# Supplementary material for: Cortical layer 6b mediates state-dependent changes in brain activity and effects of orexin on waking and sleep
Source: eLife. 2026 Sep 8;14:RP106992. doi: 10.7554/eLife.106992 (PMC13553069; doi:10.7554/eLife.106992)
Supplement: Supplementary file 2 [file elife-106992-data1.docx]

DATASET for Maijer et al., eLife

analysis: <https://doi.org/10.6084/m9.figshare.31830526>

EEG spectra: <https://doi.org/10.6084/m9.figshare.31830520>

histology: <https://doi.org/10.6084/m9.figshare.31830289>

vigilance state annotation: <https://doi.org/10.6084/m9.figshare.31375441>

| Matlab scripts and functions | | |
| --- | --- | --- |
| Resampling_Feb2022_20241027.m | | This script resamples the EEG data that have been exported from the TDT recording software. It uses the TDTSDK Matlab package that is provided by TDT. The script loops through all animals, recording days and channels, obtains the original sampling rate from the raw data tank and resamples the signals to 256 Hz. The signals are saved per animal, recording day and EEG channel. |
| BL_PSDperVS_fromunfilteredfiles_20241027.m | | This script calculates EEG spectral power density per epoch with the PWelch function in Matlab. It loads the signals that have been exported from the TDT software and resampled in Matlab. It loops through all animals and recording days individually. The signal is zero padded if it is shorter than 24 hours and the signal is cut off at 24 hours if it is longer than 24 hours. There is the option to apply a notch filter, which is specified in the top lines in the script. The result is saved in a file per animal, per recording day and per channel (frontal EEG, occipital EEG) |
| SleepEpisodes | | This function finds the number and duration of uninterrupted episodes of NREM sleep, REM sleep, and wakefulness, using preset variables such as the maximal duration of interruptions that are allowed. |
| BriefAwakenings | | This function finds the number of brief awakenings, short stretches of epochs scored as wakefulness during episodes of sleep. The maximal duration of interruptions that are allowed to still classify the sleep episode as a single episode despite the interruption (and the interruption not as a new wake episode) are specified in the top lines of the script. |
| EEG data | | |
| Baseline & sleep dep recording info | Information file that provides information on the Baseline and Sleep deprivation experiment. | |
| Orexin ICV schedule | Information file that provides information on the orexin intracerebroventricular (ICV) infusion experiment. | |
| Orexin spectra excluded animals | Lists excluded animals for spectral analysis including analysis of slow wave activity. We decided to exclude general spectral analysis, because the number of remaining animals was too low. | |
| OutputVSch | Output from SleepSign: per animal, EEG (1= frontal, 2=occipital), and per day for every epoch the annotation from manual sleep scoring and spectra exported from SleepSign (for spectral analysis, PWelch spectra calculated directly from raw data files in Matlab were used instead).   1. Baseline: “C1” etc refers to animal name codes (data was recorded in two cohorts, C and D); number “1” refers to baseline file; “eeg1” and “eeg2” to frontal and occipital EEG derivation, respectively 2. Sleep deprivation: as above; number “2” behind animal name codes refers to sleep deprivation file 3. Orexin: “F1” etc refers to animal name codes (experiment was recorded in 4 cohorts F,G,H,I); four-character code refers to infusion (either INF1-4 or a four-digit date code; key to decoding can be found in the Orexin ICV study file); “eeg1” and “eeg2” to frontal and occipital EEG derivation, respectively | |
| PwelchPSD | EEG spectral power density calculated with PWelch function in Matlab. Naming convention: no notch filter was applied, and this is also specified in the file name.   1. Baseline: C1” etc refers to animal name codes (data was recorded in two cohorts, C and D); date “0202” refers to baseline file for C cohort and “0224” refers to baseline file for D cohort, “ch1” and “ch2” to frontal and occipital EEG derivation, respectively 2. Sleep deprivation: C1” etc refers to animal name codes (data was recorded in two cohorts, C and D); date “0203” refers to sleep deprivation file for C cohort and “0225” refers to sleep deprivation file for D cohort, “ch1” and “ch2” to frontal and occipital EEG derivation, respectively | |

| Histology | |
| --- | --- |
| Analysis 2 - Densities/DCST_Drd1a-Cplx3_Cell-Densities.prism | Compares Drd1a-Cre positive cell densities across layers |
| Images | Image files as TIFFs and PNGs |

| Prism files | |
| --- | --- |
| Baseline_Sleeparchitecture | Prism file with statistical analysis of sleep architecture parametres |
