# Supplementary material for: Cortical layer 6b mediates state-dependent changes in brain activity and effects of orexin on waking and sleep
Source: eLife. 2026 Sep 8;14:RP106992. doi: 10.7554/eLife.106992 (PMC13553069; doi:10.7554/eLife.106992)
Supplement: Supplementary file 3 [file elife-106992-fig1-data1.docx]

**Figure 1 – figure supplement 1. Inter-layer pairwise comparisons**

*Method = Two-stage linear step-up procedure of Benjamini Krieger and Yekutieli; number of families = 4; number of comparisons per family = 21; significance defined at q/p < α = 0.05.*

| **Cortical layer**  **comparison:**  **Main results** | **M1** | | | **S1** | | |
| --- | --- | --- | --- | --- | --- | --- |
|  | **Predicted mean difference (LS)** | **q value** | **p value** | **Predicted mean difference (LS)** | **q value** | **p value** |
| L6b vs. L6a | 0.00065 | <0.0001 | <0.0001 | 0.000665 | <0.0001 | <0.0001 |
| L6b vs. L5 | 0.001448 | <0.0001 | <0.0001 | 0.000949 | <0.0001 | <0.0001 |
| L6b vs. L4 | 0.001461 | <0.0001 | <0.0001 | 0.000951 | <0.0001 | <0.0001 |
| L6b vs. L3 | 0.001456 | <0.0001 | <0.0001 | 0.000952 | <0.0001 | <0.0001 |
| L6b vs. L2 | 0.001456 | <0.0001 | <0.0001 | 0.000952 | <0.0001 | <0.0001 |
| L6b vs. L1 | 0.001461 | <0.0001 | <0.0001 | 0.000953 | <0.0001 | <0.0001 |
| L6a vs. L5 | 0.000798 | <0.0001 | <0.0001 | 0.000284 | <0.0001 | <0.0001 |
| L6a vs. L4 | 0.000811 | <0.0001 | <0.0001 | 0.000286 | <0.0001 | <0.0001 |
| L6a vs. L3 | 0.000806 | <0.0001 | <0.0001 | 0.000287 | <0.0001 | <0.0001 |
| L6a vs. L2 | 0.000806 | <0.0001 | <0.0001 | 0.000287 | <0.0001 | <0.0001 |
| L6a vs. L1 | 0.000811 | <0.0001 | <0.0001 | 0.000288 | <0.0001 | <0.0001 |
| L5 vs. L4 | 1.35E-05 | 0.5 | 0.8105 | 2.33E-06 | 0.5 | 0.967 |
| L5 vs. L3 | 8.59E-06 | 0.5 | 0.8787 | 3.28E-06 | 0.5 | 0.9535 |
| L5 vs. L2 | 8.59E-06 | 0.5 | 0.8787 | 3.28E-06 | 0.5 | 0.9535 |
| L5 vs. L1 | 1.35E-05 | 0.5 | 0.8105 | 4.08E-06 | 0.5 | 0.9423 |
| L4 vs. L3 | -4.91E-06 | 0.5 | 0.9305 | 9.50E-07 | 0.5 | 0.9865 |
| L4 vs. L2 | -4.91E-06 | 0.5 | 0.9305 | 9.50E-07 | 0.5 | 0.9865 |
| L4 vs. L1 | 0 | 0.5 | >0.9999 | 1.75E-06 | 0.5 | 0.9753 |
| L3 vs. L2 | 1.08E-19 | 0.5 | >0.9999 | 0 | 0.5 | >0.9999 |
| L3 vs. L1 | 4.91E-06 | 0.5 | 0.9305 | 7.95E-07 | 0.5 | 0.9887 |
| L2 vs. L1 | 4.91E-06 | 0.5 | 0.9305 | 7.95E-07 | 0.5 | 0.9887 |
| **Cortical layer**  **comparison:**  **Main results** | **V1** | | | **PFC** | | |
|  | **Predicted mean difference (LS)** | **q value** | **p value** | **Predicted mean difference (LS)** | **q value** | **p value** |
| L6b vs. L6a | 0.000724 | <0.0001 | <0.0001 | 0.000496 | <0.0001 | <0.0001 |
| L6b vs. L5 | 0.000898 | <0.0001 | <0.0001 | 0.000915 | <0.0001 | <0.0001 |
| L6b vs. L4 | 0.000899 | <0.0001 | <0.0001 | 0.000923 | <0.0001 | <0.0001 |
| L6b vs. L3 | 0.000897 | <0.0001 | <0.0001 | 0.000922 | <0.0001 | <0.0001 |
| L6b vs. L2 | 0.000897 | <0.0001 | <0.0001 | 0.000922 | <0.0001 | <0.0001 |
| L6b vs. L1 | 0.000899 | <0.0001 | <0.0001 | 0.000923 | <0.0001 | <0.0001 |
| L6a vs. L5 | 0.000174 | 0.0454 | 0.0304 | 0.000419 | <0.0001 | <0.0001 |
| L6a vs. L4 | 0.000175 | 0.0454 | 0.0296 | 0.000427 | <0.0001 | <0.0001 |
| L6a vs. L3 | 0.000173 | 0.0454 | 0.0317 | 0.000426 | <0.0001 | <0.0001 |
| L6a vs. L2 | 0.000173 | 0.0454 | 0.0317 | 0.000426 | <0.0001 | <0.0001 |
| L6a vs. L1 | 0.000175 | 0.0454 | 0.0296 | 0.000427 | <0.0001 | <0.0001 |
| L5 vs. L4 | 8.37E-07 | 0.75 | 0.9916 | 7.98E-06 | 0.5 | 0.897 |
| L5 vs. L3 | -1.43E-06 | 0.75 | 0.9856 | 6.65E-06 | 0.5 | 0.9141 |
| L5 vs. L2 | -1.43E-06 | 0.75 | 0.9856 | 6.65E-06 | 0.5 | 0.9141 |
| L5 vs. L1 | 8.37E-07 | 0.75 | 0.9916 | 7.44E-06 | 0.5 | 0.904 |
| L4 vs. L3 | -2.27E-06 | 0.75 | 0.9772 | -1.33E-06 | 0.5 | 0.9828 |
| L4 vs. L2 | -2.27E-06 | 0.75 | 0.9772 | -1.33E-06 | 0.5 | 0.9828 |
| L4 vs. L1 | 0 | 0.75 | >0.9999 | -5.41E-07 | 0.5 | 0.993 |
| L3 vs. L2 | -1.08E-19 | 0.75 | >0.9999 | -5.42E-20 | 0.5 | >0.9999 |
| L3 vs. L1 | 2.27E-06 | 0.75 | 0.9772 | 7.89E-07 | 0.5 | 0.9898 |
| L2 vs. L1 | 2.27E-06 | 0.75 | 0.9772 | 7.89E-07 | 0.5 | 0.9898 |

*Additional test details are as follows: For M1 and S1, N1 = N2 = 6 and SE of differences = 0.00005616 with DF = 112; for V1, N1 = N2 = 3 and SE of differences = 0.00007942 with DF = 112; and for PFC, N1 = N2 = 5 and SE of differences = 0.00006152 with DF = 112.*

| **Cortical layer**  **comparison:**  **Test details** | **M1** | | | **S1** | | |
| --- | --- | --- | --- | --- | --- | --- |
|  | **Predicted mean 1 (LS)** | **Predicted mean 2 (LS)** | **t value** | **Predicted mean 1 (LS)** | **Predicted mean 2 (LS)** | **t value** |
| L6b vs. L6a | 0.001461 | 0.000811 | 11.57 | 0.000954 | 0.000289 | 11.84 |
| L6b vs. L5 | 0.001461 | 1.35E-05 | 25.78 | 0.000954 | 5.00E-06 | 16.9 |
| L6b vs. L4 | 0.001461 | 0 | 26.02 | 0.000954 | 2.67E-06 | 16.94 |
| L6b vs. L3 | 0.001461 | 4.91E-06 | 25.93 | 0.000954 | 1.72E-06 | 16.96 |
| L6b vs. L2 | 0.001461 | 4.91E-06 | 25.93 | 0.000954 | 1.72E-06 | 16.96 |
| L6b vs. L1 | 0.001461 | 0 | 26.02 | 0.000954 | 9.25E-07 | 16.97 |
| L6a vs. L5 | 0.000811 | 1.35E-05 | 14.20 | 0.000289 | 5.00E-06 | 5.057 |
| L6a vs. L4 | 0.000811 | 0 | 14.44 | 0.000289 | 2.67E-06 | 5.099 |
| L6a vs. L3 | 0.000811 | 4.91E-06 | 14.35 | 0.000289 | 1.72E-06 | 5.116 |
| L6a vs. L2 | 0.000811 | 4.91E-06 | 14.35 | 0.000289 | 1.72E-06 | 5.116 |
| L6a vs. L1 | 0.000811 | 0 | 14.44 | 0.000289 | 9.25E-07 | 5.13 |
| L5 vs. L4 | 1.35E-05 | 0 | 0.24 | 5.00E-06 | 2.67E-06 | 0.04149 |
| L5 vs. L3 | 1.35E-05 | 4.91E-06 | 0.15 | 5.00E-06 | 1.72E-06 | 0.05841 |
| L5 vs. L2 | 1.35E-05 | 4.91E-06 | 0.15 | 5.00E-06 | 1.72E-06 | 0.05841 |
| L5 vs. L1 | 1.35E-05 | 0 | 0.24 | 5.00E-06 | 9.25E-07 | 0.07256 |
| L4 vs. L3 | 0 | 4.91E-06 | 0.09 | 2.67E-06 | 1.72E-06 | 0.01692 |
| L4 vs. L2 | 0 | 4.91E-06 | 0.09 | 2.67E-06 | 1.72E-06 | 0.01692 |
| L4 vs. L1 | 0 | 0 | 0.00 | 2.67E-06 | 9.25E-07 | 0.03107 |
| L3 vs. L2 | 4.91E-06 | 4.91E-06 | 0.00 | 1.72E-06 | 1.72E-06 | 0 |
| L3 vs. L1 | 4.91E-06 | 0 | 0.09 | 1.72E-06 | 9.25E-07 | 0.01416 |
| L2 vs. L1 | 4.91E-06 | 0 | 0.09 | 1.72E-06 | 9.25E-07 | 0.01416 |
| **Cortical layer**  **comparison:**  **Test details** | **V1** | | | **PFC** | | |
|  | **Predicted mean 1 (LS)** | **Predicted mean 2 (LS)** | **t value** | **Predicted mean 1 (LS)** | **Predicted mean 2 (LS)** | **t value** |
| L6b vs. L6a | 0.000899 | 0.000175 | 9.116 | 0.000923 | 0.000427 | 8.063 |
| L6b vs. L5 | 0.000899 | 8.37E-07 | 11.31 | 0.000923 | 7.98E-06 | 14.87 |
| L6b vs. L4 | 0.000899 | 0 | 11.32 | 0.000923 | 0 | 15 |
| L6b vs. L3 | 0.000899 | 2.27E-06 | 11.29 | 0.000923 | 1.33E-06 | 14.98 |
| L6b vs. L2 | 0.000899 | 2.27E-06 | 11.29 | 0.000923 | 1.33E-06 | 14.98 |
| L6b vs. L1 | 0.000899 | 0 | 11.32 | 0.000923 | 5.41E-07 | 14.99 |
| L6a vs. L5 | 0.000175 | 8.37E-07 | 2.193 | 0.000427 | 7.98E-06 | 6.811 |
| L6a vs. L4 | 0.000175 | 0 | 2.203 | 0.000427 | 0 | 6.941 |
| L6a vs. L3 | 0.000175 | 2.27E-06 | 2.175 | 0.000427 | 1.33E-06 | 6.919 |
| L6a vs. L2 | 0.000175 | 2.27E-06 | 2.175 | 0.000427 | 1.33E-06 | 6.919 |
| L6a vs. L1 | 0.000175 | 0 | 2.203 | 0.000427 | 5.41E-07 | 6.932 |
| L5 vs. L4 | 8.37E-07 | 0 | 0.01054 | 7.98E-06 | 0 | 0.1297 |
| L5 vs. L3 | 8.37E-07 | 2.27E-06 | 0.01804 | 7.98E-06 | 1.33E-06 | 0.1081 |
| L5 vs. L2 | 8.37E-07 | 2.27E-06 | 0.01804 | 7.98E-06 | 1.33E-06 | 0.1081 |
| L5 vs. L1 | 8.37E-07 | 0 | 0.01054 | 7.98E-06 | 5.41E-07 | 0.1209 |
| L4 vs. L3 | 0 | 2.27E-06 | 0.02858 | 0 | 1.33E-06 | 0.02162 |
| L4 vs. L2 | 0 | 2.27E-06 | 0.02858 | 0 | 1.33E-06 | 0.02162 |
| L4 vs. L1 | 0 | 0 | 0 | 0 | 5.41E-07 | 0.008794 |
| L3 vs. L2 | 2.27E-06 | 2.27E-06 | 1.4E-15 | 1.33E-06 | 1.33E-06 | 8.81E-16 |
| L3 vs. L1 | 2.27E-06 | 0 | 0.02858 | 1.33E-06 | 5.41E-07 | 0.01283 |
| L2 vs. L1 | 2.27E-06 | 0 | 0.02858 | 1.33E-06 | 5.41E-07 | 0.01283 |
