# Supplementary material for: Cortical layer 6b mediates state-dependent changes in brain activity and effects of orexin on waking and sleep
Source: eLife. 2026 Sep 8;14:RP106992. doi: 10.7554/eLife.106992 (PMC13553069; doi:10.7554/eLife.106992)
Supplement: Supplementary file 4 [file elife-106992-fig1-data2.docx]

**Figure 1 – figure supplement 2. Inter-region pairwise comparisons**

*Method = Two-stage linear step-up procedure of Benjamini Krieger and Yekutieli; number of families = 7; number of comparisons per family = 6; significance defined at q/p < α = 0.05.*

| **Cortical region comparison: Main results** | | **Predicted mean difference (LS)** | **q value** | **p value** |
| --- | --- | --- | --- | --- |
| **L6b** | M1 vs. S1 | 0.000507 | <0.0001 | <0.0001 |
|  | M1 vs. V1 | 0.000562 | <0.0001 | <0.0001 |
|  | M1 vs. PFC | 0.000538 | <0.0001 | <0.0001 |
|  | S1 vs. V1 | 5.50E-05 | 0.3352 | 0.4256 |
|  | S1 vs. PFC | 3.10E-05 | 0.3778 | 0.5997 |
|  | V1 vs. PFC | -2.40E-05 | 0.3865 | 0.7361 |
| **L6a** | M1 vs. S1 | 0.000522 | <0.0001 | <0.0001 |
|  | M1 vs. V1 | 0.000636 | <0.0001 | <0.0001 |
|  | M1 vs. PFC | 0.000384 | <0.0001 | <0.0001 |
|  | S1 vs. V1 | 0.000114 | 0.0175 | 0.1002 |
|  | S1 vs. PFC | -0.00014 | 0.0044 | 0.0209 |
|  | V1 vs. PFC | -0.00025 | 0.0001 | 0.0006 |
| **L5** | M1 vs. S1 | 8.50E-06 | >0.9999 | 0.88 |
|  | M1 vs. V1 | 1.27E-05 | >0.9999 | 0.8543 |
|  | M1 vs. PFC | 5.52E-06 | >0.9999 | 0.9255 |
|  | S1 vs. V1 | 4.16E-06 | >0.9999 | 0.9518 |
|  | S1 vs. PFC | -2.98E-06 | >0.9999 | 0.9597 |
|  | V1 vs. PFC | -7.14E-06 | >0.9999 | 0.9201 |
| **L4** | M1 vs. S1 | -2.67E-06 | >0.9999 | 0.9622 |
|  | M1 vs. V1 | 0 | >0.9999 | >0.9999 |
|  | M1 vs. PFC | 0 | >0.9999 | >0.9999 |
|  | S1 vs. V1 | 2.67E-06 | >0.9999 | 0.9691 |
|  | S1 vs. PFC | 2.67E-06 | >0.9999 | 0.9639 |
|  | V1 vs. PFC | 0 | >0.9999 | >0.9999 |
| **L3** | M1 vs. S1 | 3.19E-06 | >0.9999 | 0.9548 |
|  | M1 vs. V1 | 2.64E-06 | >0.9999 | 0.9695 |
|  | M1 vs. PFC | 3.58E-06 | >0.9999 | 0.9516 |
|  | S1 vs. V1 | -5.50E-07 | >0.9999 | 0.9936 |
|  | S1 vs. PFC | 3.90E-07 | >0.9999 | 0.9947 |
|  | V1 vs. PFC | 9.40E-07 | >0.9999 | 0.9895 |
| **L2** | M1 vs. S1 | 3.19E-06 | >0.9999 | 0.9548 |
|  | M1 vs. V1 | 2.64E-06 | >0.9999 | 0.9695 |
|  | M1 vs. PFC | 3.58E-06 | >0.9999 | 0.9516 |
|  | S1 vs. V1 | -5.50E-07 | >0.9999 | 0.9936 |
|  | S1 vs. PFC | 3.90E-07 | >0.9999 | 0.9947 |
|  | V1 vs. PFC | 9.40E-07 | >0.9999 | 0.9895 |
| **L1** | M1 vs. S1 | -9.25E-07 | >0.9999 | 0.9869 |
|  | M1 vs. V1 | 0 | >0.9999 | >0.9999 |
|  | M1 vs. PFC | -5.41E-07 | >0.9999 | 0.9927 |
|  | S1 vs. V1 | 9.25E-07 | >0.9999 | 0.9893 |
|  | S1 vs. PFC | 3.84E-07 | >0.9999 | 0.9948 |
|  | V1 vs. PFC | -5.41E-07 | >0.9999 | 0.9939 |

*Additional test details are as follows: For SE of differences, M1 vs S1 = 5.62E-05, M1 vs V1 = S1 vs V1 = 6.88E-05, M1 vs PFC = S1 vs PFC = 5.89E-05, and V1 vs PFC = 7.10E-05; N = 6 for M1 and S1, N = 3 for V1, and N = 5 for PFC; and DF = 112 for all comparisons.*

| **Cortical region comparison: Test details** | | **Predicted mean 1 (LS)** | **Predicted mean 2 (LS)** | **t value** |
| --- | --- | --- | --- | --- |
| **L6b** | M1 vs. S1 | 0.001461 | 0.000954 | 9.028 |
|  | M1 vs. V1 | 0.001461 | 0.000899 | 8.171 |
|  | M1 vs. PFC | 0.001461 | 0.000923 | 9.134 |
|  | S1 vs. V1 | 0.000954 | 0.000899 | 0.7997 |
|  | S1 vs. PFC | 0.000954 | 0.000923 | 0.5263 |
|  | V1 vs. PFC | 0.000899 | 0.000923 | 0.3379 |
| **L6a** | M1 vs. S1 | 0.000811 | 0.000289 | 9.295 |
|  | M1 vs. V1 | 0.000811 | 0.000175 | 9.247 |
|  | M1 vs. PFC | 0.000811 | 0.000427 | 6.52 |
|  | S1 vs. V1 | 0.000289 | 0.000175 | 1.657 |
|  | S1 vs. PFC | 0.000289 | 0.000427 | 2.343 |
|  | V1 vs. PFC | 0.000175 | 0.000427 | 3.548 |
| **L5** | M1 vs. S1 | 1.35E-05 | 5.00E-06 | 0.1514 |
|  | M1 vs. V1 | 1.35E-05 | 8.37E-07 | 0.1841 |
|  | M1 vs. PFC | 1.35E-05 | 7.98E-06 | 0.09372 |
|  | S1 vs. V1 | 5.00E-06 | 8.37E-07 | 0.06053 |
|  | S1 vs. PFC | 5.00E-06 | 7.98E-06 | 0.05059 |
|  | V1 vs. PFC | 8.37E-07 | 7.98E-06 | 0.1006 |
| **L4** | M1 vs. S1 | 0 | 2.67E-06 | 0.04754 |
|  | M1 vs. V1 | 0 | 0 | 0 |
|  | M1 vs. PFC | 0 | 0 | 0 |
|  | S1 vs. V1 | 2.67E-06 | 0 | 0.03882 |
|  | S1 vs. PFC | 2.67E-06 | 0 | 0.04533 |
|  | V1 vs. PFC | 0 | 0 | 0 |
| **L3** | M1 vs. S1 | 4.91E-06 | 1.72E-06 | 0.0568 |
|  | M1 vs. V1 | 4.91E-06 | 2.27E-06 | 0.03838 |
|  | M1 vs. PFC | 4.91E-06 | 1.33E-06 | 0.06078 |
|  | S1 vs. V1 | 1.72E-06 | 2.27E-06 | 0.007997 |
|  | S1 vs. PFC | 1.72E-06 | 1.33E-06 | 0.006621 |
|  | V1 vs. PFC | 2.27E-06 | 1.33E-06 | 0.01323 |
| **L2** | M1 vs. S1 | 4.91E-06 | 1.72E-06 | 0.0568 |
|  | M1 vs. V1 | 4.91E-06 | 2.27E-06 | 0.03838 |
|  | M1 vs. PFC | 4.91E-06 | 1.33E-06 | 0.06078 |
|  | S1 vs. V1 | 1.72E-06 | 2.27E-06 | 0.007997 |
|  | S1 vs. PFC | 1.72E-06 | 1.33E-06 | 0.006621 |
|  | V1 vs. PFC | 2.27E-06 | 1.33E-06 | 0.01323 |
| **L1** | M1 vs. S1 | 0 | 9.25E-07 | 0.01647 |
|  | M1 vs. V1 | 0 | 0 | 0 |
|  | M1 vs. PFC | 0 | 5.41E-07 | 0.009185 |
|  | S1 vs. V1 | 9.25E-07 | 0 | 0.01345 |
|  | S1 vs. PFC | 9.25E-07 | 5.41E-07 | 0.00652 |
|  | V1 vs. PFC | 0 | 5.41E-07 | 0.007616 |
