# Supplementary material for: Cortical layer 6b mediates state-dependent changes in brain activity and effects of orexin on waking and sleep
Source: eLife. 2026 Sep 8;14:RP106992. doi: 10.7554/eLife.106992 (PMC13553069; doi:10.7554/eLife.106992)
Supplement: Supplementary file 5 [file elife-106992-fig3-data1.docx]

|  | **EEG** | **F(DFn, DFd)** | **p** | **Significant posthoc comparisons** |
| --- | --- | --- | --- | --- |
| **Wake** | Frontal | F(500,700)=1.235 | p=0.0004 | 8.0-10.75, 12-12.75 Hz |
|  | Occipital | F(500,6500)=0.6897 | p>0.9999 | 9.0-13.75, 14-15 Hz |
| **NREM** | Frontal | F(500,7000)=5.566 | p<0.0001 | 3.0-67.75 Hz |
|  | Occipital | F(500,6500)=1.131 | p=0.0274 | 84.25-128 Hz |
| **REM** | Frontal | F(500,7000)=1.563 | p<0.0001 | 8.0-10 Hz, 14.75-107.5 Hz |
|  | Occipital | F(500,6500)=6.284 | p<0.0001 | 8.5-10 Hz, 69.75-72.25 Hz, 72.75-105.5 Hz, 106.75-128 Hz |

**Figure 3 – figure supplement 2. Comparison of EEG spectra from L6b silenced and control animals across the full frequency range recorded.** Comparisons were made between spectra 0.0-128 Hz in 0.25 Hz bins, after exclusion of bins 0.0-0.5 Hz and 49-51.5 Hz (electrical noise), using two-way ANOVAs. Results are shown for Genotype x Frequency interaction. Frontal EEG, controls n=7, L6b silenced n=9; Occipital EEG, controls n=6, L6b silenced n=9.
