# Supplementary material for: Cortical layer 6b mediates state-dependent changes in brain activity and effects of orexin on waking and sleep
Source: eLife. 2026 Sep 8;14:RP106992. doi: 10.7554/eLife.106992 (PMC13553069; doi:10.7554/eLife.106992)
Supplement: Peer review history

# Editor report: eLife Assessment

**DOI:** 10.7554/eLife.106992.3.sa0

**Authors:** John R Huguenard

**Affiliations:** Stanford University School of Medicine United States

**Keywords:** Important, Solid

This study offers an **important** contribution to our understanding of the role of layer 6b cortical neurons in sleep-wake regulation, providing new insight into how this understudied neural population may regulate cortical arousal via orexin signaling. The evidence supporting these findings is **solid**, although somewhat constrained by limitations in the specificity of the genetic targeting strategy. Nonetheless, the work introduces new avenues for uncovering how the classical wake-promoting peptide, orexin, exerts its effects on the cortex.

# Referee report: Reviewer #1 (Public review)

**DOI:** 10.7554/eLife.106992.3.sa1

Summary:

Meijer et al. sought to investigate the role of cortical layer 6b (L6b) neurons in modulating sleep-wake states and cortical oscillations under baseline and sleep deprived conditions and in response to orexin A and B. Using chronic EEG recordings in mice with silencing of Drd1a+ neurons (via constitutive Cre-dependent knockout of SNAP25), the authors report that while overall baseline sleep-wake architecture and response to sleep deprivation are minimal/unchanged, "L6b silencing leads" to a slowing of theta activity during wakefulness and REM sleep, and a reduction in EEG power during NREM sleep. The manuscript is well written with clarity and transparency. Although Drd1a+ neurons are not exclusive to L6b, the authors describe key future studies to identify a causal role for L6b neurons in brain state regulation. These studies contribute to a growing body of evidence that cortex-in addition to subcortical brain regions-plays a role in brain state regulation.

Strengths:

(1) The text is well written.

(2) The authors are transparent about methodological details and study limitations.

(3) The stated sleep, circadian, and orexin infusion experiments are well designed, executed, and analyzed.

Weaknesses:

(1) Outcomes are attributed to silencing cortical L6b neurons, but the genetic manipulation is not specific to L6b neurons or cortex. The authors acknowledge this as a limitation and offer targets for future studies to identify L6b neuron-specific contributions to stated outcomes that include spatially restricted manipulations.

(2) Experiments use only male mice, which limits generalizability to females.

Comments on revised version:

The authors took great care in addressing my previous comments, and I do not have any additional concerns.

# Referee report: Reviewer #2 (Public review)

**DOI:** 10.7554/eLife.106992.3.sa2

Summary:

In this manuscript, Meijer and colleagues investigated the effects of inactivation (conditional silencing) of cortical layer 6b neurons on sleep-wake states and EEG spectral power under the following three conditions: during natural sleep-wake states, after sleep deprivation, or after intracerebroventricular administration of orexin A and B. The authors report that silencing of L6b neurons did not have a significant effect on the total time spent in sleep-wake states, duration or number of state epochs, or the response to sleep deprivation. However, silencing of L6b neurons did slow down theta-frequency (6-9 Hz) during wake and REM sleep, and reduced the total EEG power during NREM sleep. Infusion of orexin A in the mice in which cortical layer 6b neurons were inactivated produced an increase in wakefulness. A similar effect was observed after infusion of orexin A in the mice in which these neurons were not silenced, but the effect (i.e., increase in wakefulness) was of a smaller magnitude. Silencing of cortical layer 6b neurons attenuated the effect of orexin B in increasing theta activity, as was observed in the control mice. The authors conclude that the cortical neurons in layer 6b play an essential role in state-dependent dynamics of brain activity, vigilance state control and sleep regulation.

Strengths:

- A focus on cortical layer 6b neurons, which is an understudied neuronal population, especially in the context of brain and behavioral state transitions.

- The authors used a well-established mouse model to study the effect of inactivation of cortical layer 6b neurons.

Weaknesses:

- Although the authors used a highly selective approach to silence layer 6b neurons, the observed changes in EEG oscillations cannot be solely attributed to layer 6b neurons because of the ICV route for orexin administration.

- The rationale for using only male rats is not provided.

Comments on revised version:

The authors have addressed my concerns.

# Author comment: Author response

**DOI:** 10.7554/eLife.106992.3.sa3

**Authors:** Elise J Meijer, Marissa Mueller, Lukas B Krone, Tomoko Yamagata, Anna Hoerder-Suabedissen, Sian Wilcox, Hannah Alfonsa, Atreyi Chakrabarty, Luiz Guidi, Peter L Oliver, Vladyslav V Vyazovskiy, Zoltan Molnar

**Affiliations:** University of Oxford Oxford United Kingdom; University of Oxford Oxford United Kingdom; University of Oxford Oxford United Kingdom; University of Oxford Oxford United Kingdom; University of Oxford Oxford United Kingdom; University of Oxford Oxford United Kingdom; University of Oxford Oxford United Kingdom; University of Oxford Oxford United Kingdom; University of Oxford Oxford United Kingdom; Mary Lyon Centre at MRC Harwell Oxfordshire United Kingdom; University of Oxford Oxford United Kingdom; University of Oxford Oxford United Kingdom

The following is the authors’ response to the original reviews.

**Public Reviews:**

**Reviewer #1 (Public review):**

(1) All outcomes are attributed specifically to L6b neurons, but the genetic manipulation is not specific to L6b neurons. The authors acknowledge this as a limitation, but in my view, this global manipulation is more than a limitation - it affects the overall interpretations of the data. The Hoerder-Suabedissen et al., 2018 paper shows sparse, but also dense, expression of Drd1a+ neurons in brain regions outside of the L6b. Given this issue, the results are largely overstated throughout the paper.

We appreciate the reviewer’s careful reading and concern that some of our statements may have overstated the implications of our data. The Drd1a Cre mouse model used (FK164) has a relatively selective expression of Drd1a Cre in cortex, but indeed some expression is seen subcortically. This is an acknowledged limitation which is now explicitly addressed in the revised manuscript.

(2) It is not clear to me that the "silencing" of Drd1a+ neurons was verified.

In our previous publications, we showed confirmation of the loss of regulated synaptic vesicle release from the Cre-positive neuronal population (Marques-Smith et al., 2016; Hoerder-Suabedissen et al., 2018; Messore et al., 2024). This has now been described in the revised manuscript.

(3) There were various discrepancies (and potentially misattributions) between the stated significant differences in Supplementary Table T1 data and Figure 3a & S2 spectral plots. This issue makes it difficult to effectively evaluate the main text and stated outcomes.

We thank the reviewer for their careful attention to the statistical analyses and for noting the inconsistencies in how the results of the spectral analysis were presented: in the text we described two-way ANOVAs with according posthoc tests but in the figures significance markers were positioned based on multiple t tests. We have now carefully revised the spectral results and implemented a consistent approach in statistical reporting and spectral plots. We have updated Supplementary Table T1, Figure 3a and S2 to ensure that all statistics are presented consistently throughout the manuscript, i.e. with two-way ANOVAs and accompanying posthoc tests. Please note that we performed all spectral analyses in the range between 0.5 and 128 Hz (excluding the range between 49-51.5 Hz due to electrical noise from the power grid) but only plot the range between 0.5-30 Hz as the spectral bands most relevant for sleep neurophysiology are contained in this range.

Related, the authors stated that post hoc comparisons of EEG spectral frequency bins were not corrected for multiple testing. Instead, significance was only denoted if changes in at least two consecutive frequency bins were significant. However, there are multiple plots in which a single significance marker is placed over an isolated bin (i.e., 4c, 6, S5, S6). Unless each marker is equivalent to 2 consecutive frequency bins, these markers should be removed from the plots. Otherwise, please define the frequency and size of these markers in the main text.

In line with the previous comment, we have adjusted markers to reflect the results from posthoc tests after two-way ANOVAs.Please note that Figure 6 and the related supplementary figures S5 and S6 have now been removed from the manuscript, as careful re-analysis indicated that the sample size was too low to support a strong conclusion regarding the comparison of orexin effects between genotypes. We stated in the text that we would only include posthoc significance when at least two consecutive bins were significant, but this was indeed not supported in our figure, where each marker reflects one 0.25 Hz bin. We have now adjusted our code to ensure that only markers are plotted when at least two consecutive bins are significant in bin-wise posthoc comparisons.

(4) A rainbow color scale, as in Figure 3, we've now learned, can be misleading and difficult to interpret. The viridis color scale or a different diverging color scale are good alternatives.

Thank you for pointing this out, we have adjusted the colour scale.

(5) How much time elapsed between vehicle/orexin A & B infusions?

There were 2-4 non-infusions days between infusions. We have added this information to methods.

(6) For Figure 6, there are statistical discrepancies between the main text and the plots (pg. 10):

(a) The text claims post hoc differences for relative ORXA frontal EEG, but there are no significance markers on the plot.

(b) The text states that there were no post hoc differences for the relative ORXA occipital EEG, but significance markers are on the plot.

(c) The main test for the relative ORXB frontal EEG was not significant, but there are post hoc significance markers on the plot.

(d) For relative ORXB occipital EEG, there are significant markers on the plot outside of the stated range in the text.

We agree with the reviewer, and we decided to exclude this figure from the manuscript as the sample size for some key comparisons was too low to support any strong conclusions and therefore presenting this analysis is potentially misleading. We explain the rationale for excluding this analyses in the revised manuscript.

(7) Some important details are only available in figure captions, making it difficult to understand the main text. For example, when describing Figure 3c in the main text on page 7, it is not clear what type of transitions are being discussed without reading the figure caption. Likewise, a "decrease," "shift," and "change" are mentioned, but relative to what? Similar comment for the EEG theta activity description on pages 7 - 8. Please add relevant details to the main text.

We have adjusted the wording in the main text to reflect more precisely which comparisons are shown in the figures.

(8) Statistical comparisons for data in Figure 3e, post hoc analyses for data in Figure S7a-b REM data, and post hoc analyses for Figure S7c (not b) occipital EEG should be included to support differences claims. Please denote these differences on the respective plots.

Please note that the previously named Supplementary Figures S5 and S6 have been removed from the manuscript, and that the Supplementary Figure S7 in this comment refers to the figure currently named Supplementary Figure S5.

We have added the statistical comparisons for Figure 3e, Supplementary Figure S5A and Figure S5b to the results section. In Figure S5c, there was an overall genotype difference, but there was no significant time x genotype interaction, so we have not performed posthoc tests and did not plot posthoc significance markers for this figure. We have adjusted the wording in the results section to make this clearer. We have adjusted the reference to the figure S5c which was incorrect, thank you for your careful attention.

(9) In the subsection titled "Layer 6b mediates effects of orexin on vigilance states (pg. 8)," there does not seem to be any stated differences between control and L6b silenced mice. A more accurate subtitle is needed.

We agree with the reviewer and the title of this sub-section has now been changed accordingly.

**Reviewer #2 (Public review):**

Weaknesses:

(1) Although the authors used a highly selective approach to silence layer 6b neurons, the observed changes in EEG oscillations cannot be solely attributed to layer 6b neurons because of the ICV route for orexin administration.

We thank the reviewer for this important comment. The ICV route of orexin administration cannot guarantee that only cortical Drd1a-Cre–expressing neurons are reached by orexin, and the Drd1a-Cre driver line is highly selective but not entirely specific for layer 6b neurons (see also response to reviewer #1, comment 1). We have therefore changed the wording of the stated effects and addressed this consideration in the Limitations section of the manuscript. Please note that, as mentioned above, Figure 6 has now been excluded from the manuscript.

(2) The rationale for using only male rats is not provided.

We thank the reviewer for highlighting this omission. We now provide the rationale for using only male mice in the methods section as follows: “In the current study, only male mice were used, because our experimental protocol precluded the possibility of accurately monitoring the oestrous cycle, which has marked effects on brain activity, arousal and vigilance states. We therefore decided to use male mice only for the current study but are planning to use both sexes in future work.”

**Recommendations for the authors:**

**Reviewer #1 (Recommendations for the authors):**

(1) Better descriptions of L6b connectivity will improve clarity in the second paragraph of the Introduction (pg. 3). For example, it is not explicitly stated that L6b projects to L5 before the authors describe L5. Therefore, the L5 description seems irrelevant.

We thank the reviewer for this request for clarification. We mention the connectivity between L6b and L5 because L5 pyramidal neurons have recently been found to play a key role in sleep-wake regulation (Krone et al., Nat. Neurosci. 2021; Honjo et al., 2025; Wasilczuk et al, 2025; Krone et al., 2025). We have now amended the corresponding section of the introduction to emphasise the potential functional relevance of this connection as follows:

“L5, the major output layer of the cortex, is also bidirectionally communicative with higher order thalamic nuclei (Hoerder-Suabedissen et al., 2018) as well as layer 5 pyramidal neurons (Zolnik et al., 2024). Since several subtypes of L5 pyramidal neurons have recently been shown to play important roles in distinct aspects of sleep-wake regulation (Krone et al., 2021, 2025; Hong et al. 2023; Wasilczuk et al. 2025; Honjo et al., 2025; Chouafeev et al., 2025); depth of anaesthesia (Wasilczuk et al. 2025), and the influence of stress on sleep (Chouafeev et al. 2025) the projections of orexin-sensitive L6b to L5 pyramidal neurons may be a key circuitry in the top-down regulation of brain states.”

(2) There are plots where the y-axis tick label appears to be offset from the tick mark (4a, S5b, S6a).

Thank you for spotting this graphical issue. We have removed the y-axis tick labels from Figure 4a to avoid confusion. Please note that we decided to remove Figure S5 and Figure S6, because after careful re-analysis we concluded that the group size was too small to draw conclusions on orexin spectra and that any results could be potentially misleading.

(3) The 2-h time constant, I believe, is depicted in Figure 4H (not 4G).

Thank you for spotting this. We have corrected the figure legends accordingly and double-checked that Figure 4G depicts the 2-h time constant and Figure 4H the 6-h time constant.

(4) "...although there was an indication of a higher absolute theta-peak power in layer 6b silenced mice (Figure S6)," pg. 10. It is not clear to me how the data lead to this conclusion.

Thank you for identifying this inconsistency, which resulted from a preliminary statistical analysis subsequently corrected. We have now improved the statistical analysis of spectral data (for more details see comments to both reviewers in public response) and removed this statement, which in fact is no longer supported by the data.

(5) Exclusion of female mice is not listed as a limitation.

We now discuss this limitation as follows:

“In the current study, only male mice were used, because our experimental protocol precluded the possibility of accurately monitoring the oestrous cycle, which has marked effects on brain activity, arousal and vigilance states. We therefore decided to use male mice only for the current study but are planning to use both sexes in future work.”

(6) A brief description of why Cplx3 and Tbr1 antibodies are being used will be helpful to include in the Methods (pg. 21) in addition to what is in the figure caption.

We have added the following information to the methods section to clarify why we used these two antibodies: “rabbit α-Cplx3 to distinguish between L6a and L6b” “mouse α-Tbr1 to identify the L5-6 boundary”

(7) Including a label/title for the Figure 2c spectral plots will be helpful. It is not immediately clear if these are light period & dark period data or frontal & occipital data.

Thank you for pointing this out, we have updated the figure legend to clarify what is shown on this Figure

Similar comments for S2 and S3a plots. Including a state label on the plots will be helpful in addition to the caption description.

We have now added the state labels for Figure panels S2 and S3a for improved clarity.

**Reviewer #2 (Recommendations for the authors):**

This is a soundly conducted and well-written study that enhances our understanding of the cortical control of states of consciousness. I do not have any major concerns, but would like the authors to consider some alternate possibilities as suggested in my comments below:

We thank the reviewer for this positive assessment of our manuscript and the helpful suggestions.

(1) Given that the inactivation of layer6b neurons did not affect the time spent in sleep-wake states, to me it appears that these neurons likely have a role in creating the background neural conditions/oscillations supportive of an activated state rather than a direct role in behavioral state control.

We completely agree with the reviewer and have made the wording more consistent throughout the manuscript, now using “brain state control” rather than “behavioural state control” to clarify that the main effect observed in the L6b-silenced mouse model is a change in spectral characteristics reflecting brain oscillations, rather than effects on vigilance states, which were modest.

(2) Does the observed shift in REM sleep-related theta-peak frequency in the occipital derivation suggest changes in local neural processes, or could it be just a matter of better signal detection because theta is most prominent at or around the hippocampal region, which is approximately the location of occipital electrodes in this study.

The source of the shift in REM sleep–related theta peak frequency in the occipital derivation cannot be established with EEG recordings alone. Additional intracortical or intrahippocampal recordings would be necessary to distinguish between the two possible explanations proposed by the reviewer. We have discussed this further in the revised manuscript.

(3) Orexinergic system innervates multiple subcortical sites and widely covers the cortex too, because of which the effect of ICV orexins cannot be attributed to just layer6b neurons as described in the manuscript ("Layer 6b mediates effects of orexin on brain activity.").

We agree with the reviewer that this is a limitation. We have now adjusted the subtitle of the paragraph describing the results from the ICV administration of orexin and further mention this important consideration in the ‘limitations’ section of the discussion.

(4) While the current study is focused on sleep-wake mechanisms, the findings reported here have much broader implications for behavioral and/or brain state arousal and provide a mechanistic bridge between different states of consciousness, including general anesthesia. Therefore, the authors may consider tying these findings with the recent work on the role of the prefrontal cortex in arousal from general anesthesia and slow-wave sleep (PMID: 35436248, PMID: 29937348, PMID: 33328847).

We thank the reviewer for this excellent recommendation. We are now citing these papers in the revised manuscript.

(5) It's up to the authors, but I do not see the need for the section on Clinical Implications. It's very speculative, and it makes the entire discussion section heavy.

We have considerably shortened the discussion of potential clinical implications to make the manuscript more concise.

(6) Figure 1: It's difficult to compare the EEG power the way figures are set up right now. I think it would enhance clarity if the authors separate the plots based on state and show power from the control and silenced neuronal group in the same plot. Also, the colors are too similar (essentially a shade of green/blue) to provide effective visual resolution. This is especially true in panel d. Please consider changing the color scheme.

This comment seems to refer to Figure 2 and subsequent figures with analysis of vigilance states and EEG spectra (Figure 1 contains histological images). We have selected the colour scheme for colour-blind individuals. Therefore, the main difference is in the saturation, not the colour of the plots. We have tested the visibility of the colour scheme on a high-resolution screen with the original image files and can reassure the reviewer that the genotype differences, which are slightly blurred in the reduced-resolution figures provided within the combined text file for the review process, are easily distinguishable in the final figure quality.

(7) I don't understand the y-axis scale in Figure 1. How can this be 500% and if it is, then 500% of what?

This comment also seems to refer to the analysis of slow wave activity (SWA) in Figure 2 rather than to Figure 1 (histology figure). The percentage of SWA is normalised to the average SWA across the recording. Since NREM sleep is characterised by considerably higher SWA than wakefulness and REM sleep, the level of SWA during NREM sleep is in the range of 200-300%, and can be even higher after long wake episodes which are followed by a rebound of NREM sleep SWA. Hence, the upper limit of the y-axis in these (and subsequent) plots of SWA is 500% (of the average SWA). We have amended the figure legend to clarify that SWA is presented here as percentage of average SWA across the recording.
